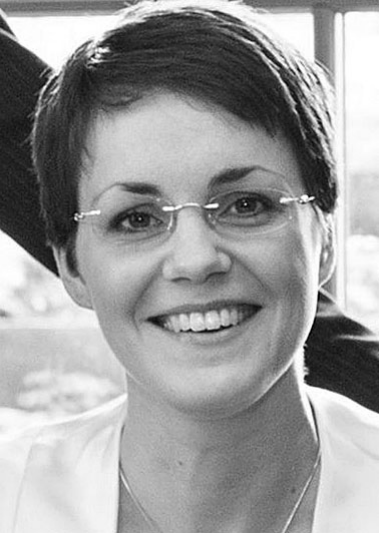# Iron Catalyzed Dehydrocoupling of Amine‐ and Phosphine‐Boranes

**DOI:** 10.1002/ijch.201700018

**Published:** 2017-07-27

**Authors:** Nathan T. Coles, Ruth L. Webster

**Affiliations:** ^1^ Department of Chemistry University of Bath Claverton Down Bath UK. BA2 7AY.

**Keywords:** homogeneous catalysis, heterogeneous catalysis, main group elements, iron, dehydropolymerization

## Abstract

Catalytic dehydrocoupling methodologies, whereby dihydrogen is released from a substrate (or intermolecularly from two substrates) is a mild and efficient method to construct main group element‐main group element bonds, the products of which can be used in advanced materials, and also for the development of hydrogen storage materials. With growing interest in the potential of compounds such as ammonia‐borane to act as hydrogen storage materials which contain a high weight% of H_2_, along with the current heightened interest in base metal catalyzed processes, this review covers recent developments in amine and phosphine dehydrocoupling catalyzed by iron complexes. The complexes employed, products formed and mechanistic proposals will be discussed.

## 1 Introduction

### 1.1 Background

**Scheme 1:**
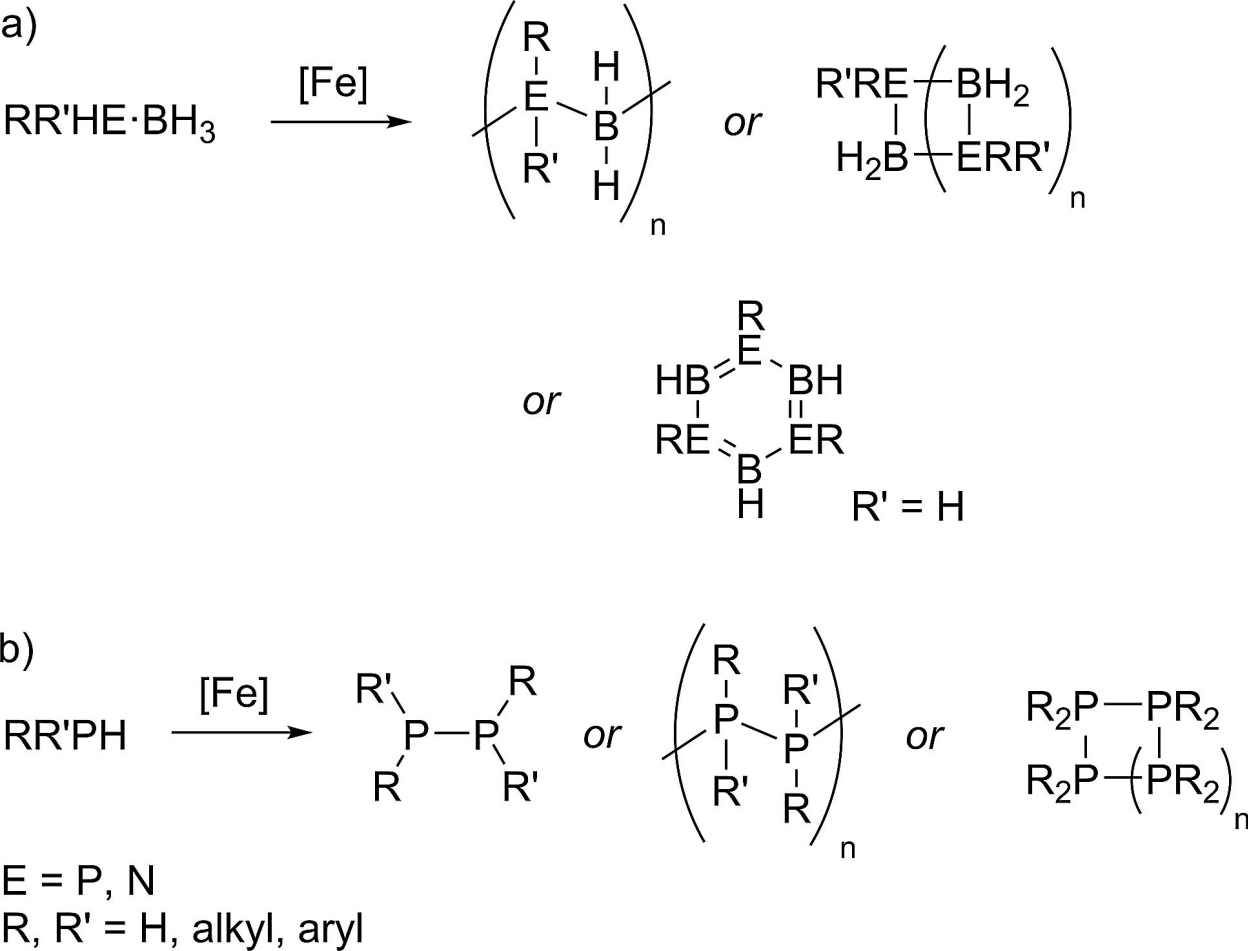
General scheme for a dehydrocoupling reaction.

Dehydrocoupling (DHC) is the term commonly used to describe the process where a bond is formed between two compounds with the loss of dihydrogen. Focusing specifically on the DHC of main group compounds, there are many examples of this type of reaction in the literature for both homo‐ and hetero‐DHC and several comprehensive reviews covering the catalysts employed, products formed, reaction mechanism and applications are available in the literature.^1^ There is a large amount of research into this area because compounds like ammonia‐borane are postulated to be excellent hydrogen storage compounds.^2^ The reason ammonia‐borane is a desirable storage material is due to it having a high weight percentage of hydrogen (19.6 %), it is also stable in air and is not highly flammable, meaning it is easy to handle and store. However, current fuel supply systems rely on the transport of liquid fuels (e. g. petroleum), an ideal scenario would be to develop a liquid hydrogen storage material that can be easily regenerated;^3^ the fact that ammonia‐borane is a solid means that in order to meet this requirement solvent is needed and thus storage efficiency is reduced. There are other issues surrounding the use of ammonia‐borane as a hydrogen source: these compounds usually require vigorous heating to dispel H_2_ and there is not a simple solution to regenerate spent fuel, as hydrogenation of the spent fuel is not energy efficient due to the stability of the products. Finding an efficient catalytic method for DHC is thus an important area of research because this would allow facile DHC at lower temperature and give insight into how to undertake facile fuel regeneration (i. e. using reverse reaction studies). Due to the importance of the wider area of group 15‐borane DHC, the focus of this review will be on the hetero‐DHC of amine‐boranes and phosphine‐boranes along with homo‐DHC of phosphines using iron catalysis (Scheme 1).

As has been reported, a large number of the examples of dehydrocoupling found in the literature use precious metals (for example, Ru, Rh, Ir and Pt have commonly been used).1p,1q,1u,1w However, precious metals in comparison to iron are much more expensive, which is linked to abundance, meaning eventually new methods for catalysis using more abundant metals will need to be found, hence the growing focus on the use of iron catalysis research. Iron does have its drawbacks; one reason why iron chemistry is under explored is due to a large number of the complexes that are synthesized being paramagnetic. This leads to study by NMR spectroscopy being extremely difficult: the signals are paramagnetically shifted, whilst broadening of signals for substrates and products often limits *in situ* study of catalytic reactions involving high loadings of catalyst. This makes understanding the mechanism of how these catalysts work challenging, which is a vital component in being able to develop more new catalysts with greater effectiveness. Another downside to iron catalysts is that they tend to be less active than a noble metal catalyst used in the same transformation. This means that turnover numbers (TON) and turnover frequency (TOF) tend to be lower, leading to a greater catalyst loading being required for the same output of product. However, this means that there is immense potential for the development and mechanistic study of new, highly active transformations involving iron catalysts.

### 1.2 Early Examples of Dehydrocoupling

**Scheme 2:**
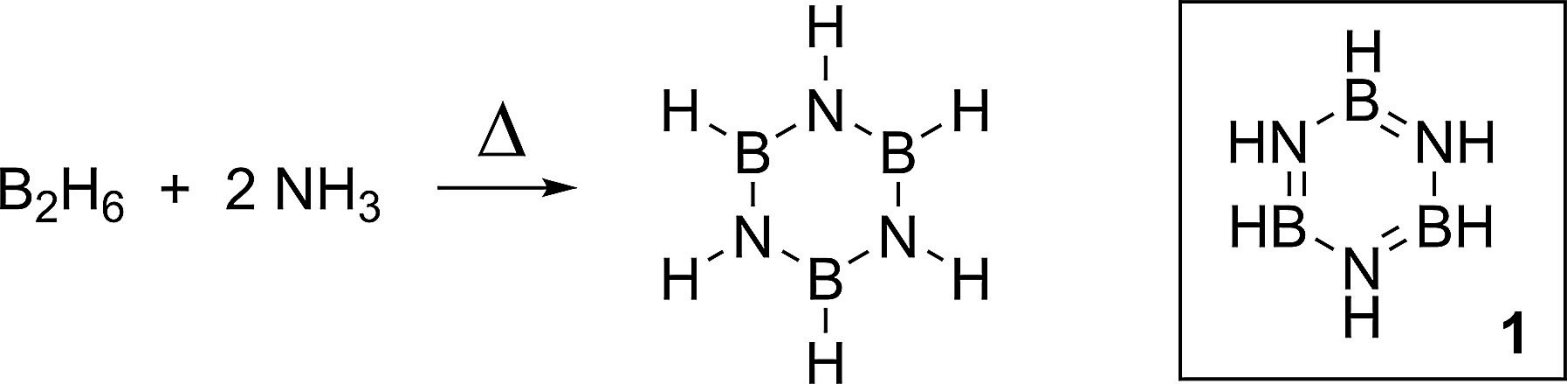
Thermally‐induced dehydrocoupling of ammonia and diborane led to borazine, which is depicted using Stock's original representation of the molecule. Insert: formal structure of borazine (**1**).

The DHC of amine‐boranes has been known since the 1920’s when Stock and Pohland synthesized borazine (**1**) *via* thermal DHC by mixing ammonia and diborane and heating at high temperatures (Scheme 2).^4^ Due to the fact that this compound is isoelectronic with benzene, which was already well studied, further work by Stock set out to understand the structure of borazine, which has similar bond lengths to benzene (with benzene's bond lengths in the region of 1.40 Å (C−C) and 1.09 Å (C−H) compared to those in borazine of 1.44 Å (B−N); 1.26 Å (B−H); 1.05 Å (N−H)).^5^ Both are also liquids at room temperature.^6^ This pioneering work set in place modern DHC chemistry and the study of properties and applications for these novel inorganic compounds.

**Figure 1:**
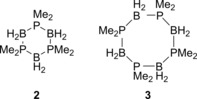
Structure of the compounds synthesized by Burg and Wagner.

In comparison, the first example of the DHC of phosphine‐boranes was not achieved until 1940 by Gamble and Gilmont.^7^ As Stock had previously shown that borazine could be formed by diammonia‐diborane (B_2_H_6_⋅2NH_3_) followed by heating, the authors tried a similar reaction with diphosphine‐diborane, B_2_H_6_⋅2PH_3_. Rather than forming a compound analogous to borazine they reported a non‐volatile residue that formed along with the release of H_2_. This would suggest DHC had taken place, most likely producing some form of polymer, however they were unable to characterise the solid obtained. It was not until 1953 that a DHC reaction of phosphine‐boranes was characterized, where dimethylphosphine‐borane was cyclized.^8^ Burg and Wagner reported cyclic products **2** and **3** (Figure 1), as well as a material that they attributed as polymeric but again this solid was not fully characterized.

## 2 Amine‐borane Dehydrocoupling

**Scheme 3:**
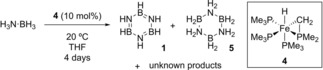
Conditions used by Baker and co‐workers to dehydrocouple ammonia‐borane using **4**, showing the original proposed product distribution.

**Figure 2:**
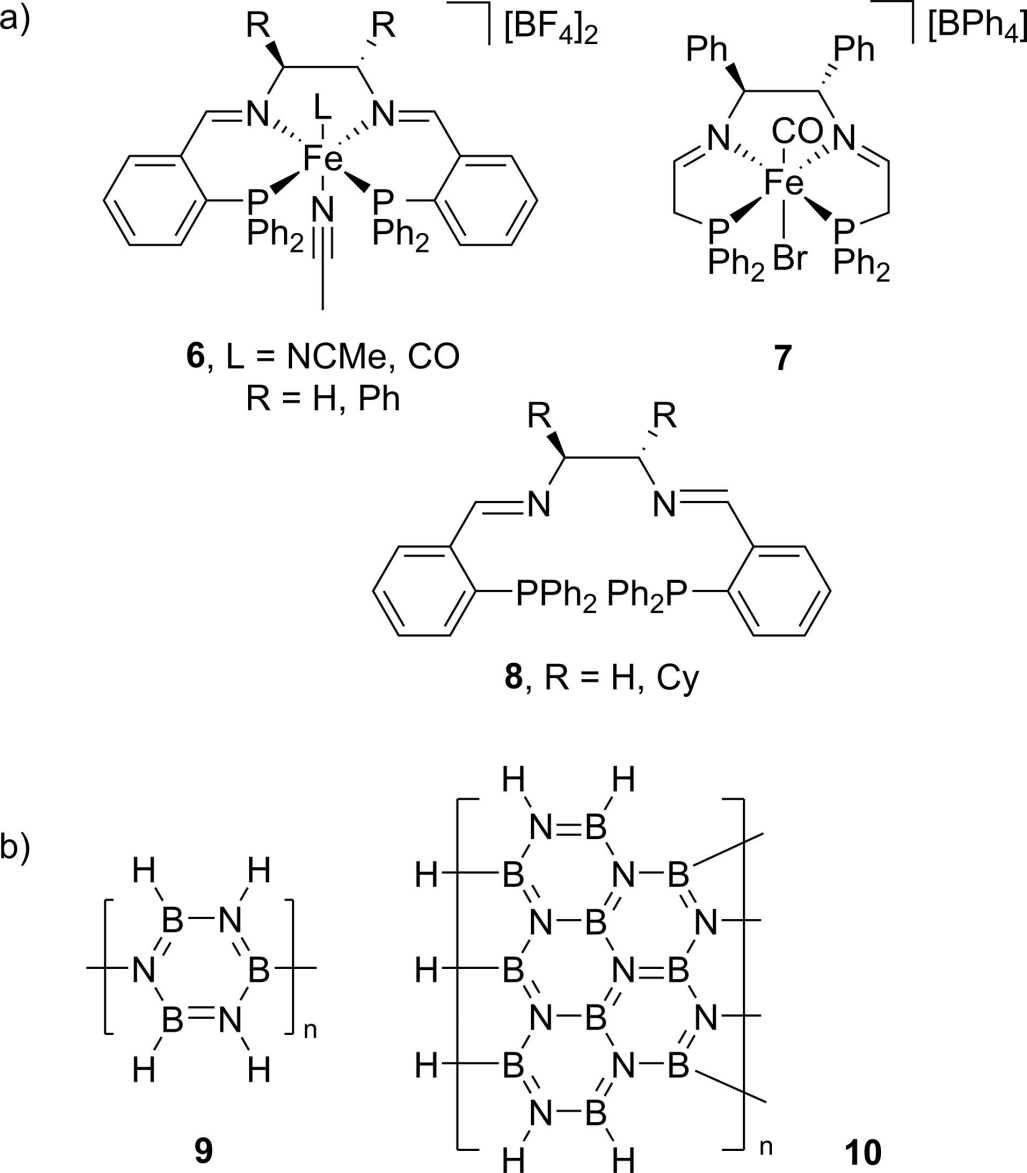
a) Structure of the pre‐catalysts and ligands used by Sonnenberg and Morris, where the iron complex of **8** was prepared *in situ* using FeBr_2_ or [Fe(H_2_O)_6_][BF_4_]_2_. b) Examples of the types of poly(borazylene) (**9**) and cross‐linked poly(borazylene) (**10**) structures that can be formed during catalysis.

The first example of an iron catalyst to be used for the DHC of an amine or phosphine‐borane was reported in 2007 by Baker and co‐workers.^9^ In the supporting information they detail the reaction of an iron hydride complex (**4**, Scheme 3) with ammonia‐borane. The reaction showed promise as DHC took place at room temperature, but in 24 hours the reaction led to a complex mixture of borazine (**1**), cyclotriborazane (**5**) along with starting material, ligand as the Lewis acid adduct (Me_3_P⋅BH_3_) and other unassigned compounds. A more recent study from Baker,^10^ that accounts for the unknown species, has identified that a key intermediate in ammonia‐borane DHC is cyclic aminoborane tetramer, B‐(cyclotriborazanyl)amine‐borane (**19**, *see* Scheme 6), which, for a range of metal catalysts was transformed into borazine and polyborazylene (see Figure 2 for typical structures). Although no mechanistic study was reported in the original 2007 paper, the authors mention the formation of a black precipitate. This could suggest the catalyst is heterogeneous but the catalyst does become deactivated with no further reaction observed when this precipitate is decanted, washed and a further portion of H_3_N⋅BH_3_ is added. This would indicate the precipitate is a by‐product of deactivation.

As a slight digression, in the following two examples from Xu^11^ and Morris,^12^ the focus is on dehydrogenation of amines‐boranes *via* catalytic methods for hydrolysis and O−B bond formation (or O−B and N−B bond formation in the case of Morris’ work), which then leads to H_2_ release. Xu and co‐workers have shown that iron nanoparticles give remarkably quick dehydrogenation of ammonia‐borane.^11^ Interestingly, it was shown that if the nanoparticles were pre‐synthesized, by reduction of FeSO_4_ with NaBH_4_ followed by the addition of substrate, the reaction was 20 times slower than if the nanoparticles were generated *in situ,* which was achieved by adding all reagents simultaneously and shaking vigorously. The latter was rapid with complete dehydrogenation of substrate being achieved in 8 minutes. Both types of nanoparticle were prepared from the same stoichiometries of reagents: 1 equivalent ammonia‐borane, 0.12 equivalents FeSO_4_ and 0.16 equivalents NaBH_4_. X‐ray diffraction patterns suggested that the pre‐prepared nanoparticles were crystalline, whereas those generated *in situ* were amorphous and zero valent in nature. When dispersed in aqueous solution it was found that the crystalline nanoparticles were agglomerated, potentially having fewer active sites than the nanoparticles generated *in situ,* which formed a suspension. This study was primarily interested in the release of H_2_, so there is no mention of what the products of DHC were for this system. Based on research from Morris (*vide infra*) borates are likely to form since water is the reaction solvent and therefore the focus of this study is more directed towards H_2_ release, rather than the preparation of N−B containing products. Nonetheless it would be interesting to compare the product distribution for pre‐synthesized versus *in situ* nanoparticle synthesis.

In a comprehensive mechanistic study, Sonnenberg and Morris also detailed evidence for DHC of amine‐boranes by iron nanoparticles formed from iron pre‐catalysts (**6**, **7** and **8** (with FeBr_2_ or [Fe(H_2_O)_6_][BF_4_]_2_ added *in situ)*, Figure 2a).^12^ Focusing specifically on catalytic DHC of ammonia‐borane, across the range of pre‐catalysts tested, DHC could be performed at room temperature or below (i. e. 2 °C) using a co‐catalytic amount of KO^t^Bu, where the catalyst : base : substrate loading was often of the order of 1 : 9 : 42. The product obtained depended on the solvent used; ^i^PrOH gave a high yield of H_2_ (often >2.5 equivalents in 1 hour) with B(O^i^Pr)_3_ being observed as the product. Interestingly, no reaction was observed when MeOH or H_2_O was used. With THF as the solvent multiple species were observed by ^11^B NMR, assigned as **1**, oligomeric or cross‐linked poly(borazylene) (Figure 2b) and in the case of pre‐catalyst **6**, where L=CO and R=H, a peak assigned to cyclotriborazane (**5**) was also observed. It was found that the bulkiness of the ligand and Fe precursor had little effect on initial rates. The authors also showed that use of a sub‐catalytic quantity of ligand, used to prepare the catalyst *in situ,* led to similar catalytic activity: if the reaction had been homogeneous in nature a co‐catalytic loading of ligand would be necessary for good activity. These observations, coupled with electron microscopy studies and the fact that the reaction halted upon addition of CO (which coats the surface of the nanoparticles preventing reactivity), provided substantial evidence that nanoparticles were indeed responsible for DHC catalysis. Although initial turnover frequencies are high, of the order of 3.66 H_2_ released per second, the overall turnover number (TON) was modest at 154.

**Scheme 4:**
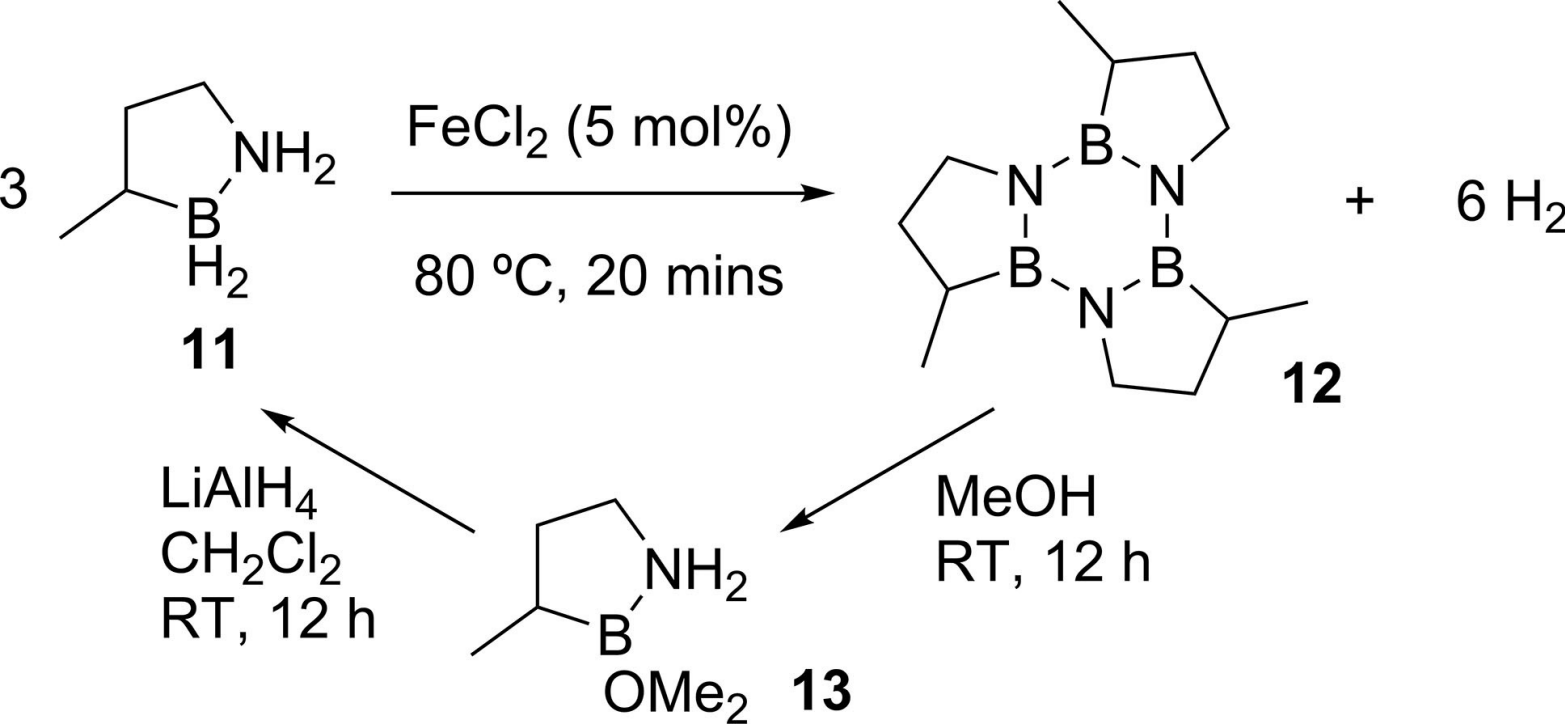
The liquid H_2_ storage system developed by Liu and co‐workers.

In 2011 Liu and co‐workers designed an H_2_ storage system where release of the gas was catalyzed by the addition of simple, cheap metal salts such as FeCl_2_ (Scheme 4).^13^ The benefits of using BN‐methylcyclopentane (**11**) as the hydrogen storage material is that it is a liquid at room temperature (and the organic product, **12**, is liquid at 28–30 °C^14^), it is also air and moisture stable and unlike other liquid hydrogen storage materials such as formic acid2o, 15 and hydrazine‐hydrate2i,2o, 16 it does not release side‐products which are likely to poison fuel cells. This system gives promise for DHC as a means to produce hydrogen from a liquid fuel source because the reaction can be carried out in neat substrate forming a liquid product, meaning issues from phase changes are avoided. This is also the first example of the simplest iron salts being used to catalyze a DHC reaction without being functionalized into a more elaborate iron complex first. The reaction also proceeded well with other simple metal chlorides (CoCl_2_, NiCl_2_, CuCl_2_ and FeCl_3_), but metal bromides were found to be the most active with 76 % conversion to **12** being achieved in five minutes using NiBr_2_ and CuBr. Focusing on the proficiency of FeCl_2_, the reaction is believed to be heterogeneous in nature as the authors reported the formation of a black precipitate during the course of the reaction. The study showed that there was little degradation of the active catalyst with three sequential additions of fuel being dehydrocoupled with similar efficacy. As a proof‐of‐concept, **11** was regenerated from **12** *via* **13** using methanol then LiAlH_4_.

Solid state DHC is somewhat unexplored compared to solution phase methodologies. To address this Chen and co‐workers used their co‐precipitation method, which follows the basic principles of the incipient wetness method, to prepare an FeB nanoalloy from FeCl_3_ and NH_3_⋅BH_3_. The active nanoalloy was shown to form by reduction of the Fe(III) starting material by ammonia‐borane and analysis data supported the formation of the FeB nanoalloy. This new heterogeneous catalyst, which displayed uniform particle size in the region of 2 to 5 nm, was then employed as a catalyst for DHC.^17^ Ammonia‐borane DHC under neat, catalyst free conditions generates amorphous materials. In comparison, the nanoalloy formed crystalline poly(aminoborane) yielding 1 equivalent H_2_ at 60 °C and 1.5 equivalents H_2_ at 100 °C and importantly the yield of NH_3_ was below the detection limit and the quantity of **1** formed, which is often a side‐product, was reduced. Although carried out on a model gas‐phase system, computational studies supported the likelihood of a Ziegler‐Natta chain‐growth mechanism.

**Scheme 5:**
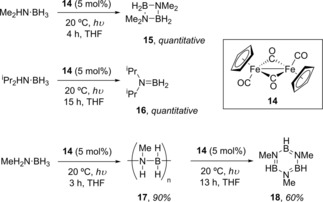
Products of various amine‐borane DHC reactions catalyzed by **14**. The reaction employs a medium pressure Hg lamp.

**Scheme 6:**
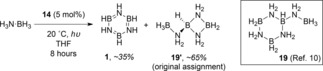
Ammonia‐borane forms a mixture of cyclic products.

Not only have Manners and co‐workers used iron Cp complexes for DHC of phosphine‐boranes (*vide infra*),^18^ but they have also used similar structures coupled with photocatalysis to perform DHC with amine‐boranes.^19^ This method for DHC is very inexpensive as the pre‐catalyst employed (**14**, Scheme 5) is commercially available and leads to high conversions with moderate to high selectivity of products, dependent on the substrate used. Interestingly, when MeNH_2_⋅BH_3_ is employed the poly(aminoborane) **17** is formed after 3 h of irradiation with a high *M*_n_ of 117,700 g mol^−1^ and moderate PDI of 1.83. However, irradiation using a medium pressure Hg lamp for a further 13 h leads to further dehydrogenation and thus decomposition of the polymeric material to form the cyclic borazine **18**. Another reaction of note is the reaction with ammonia‐borane, which had a near quantitative conversion at 20 °C after three hours and also showed a moderate selectivity for, what was originally assigned as cyclolinear trimer B‐(cyclotriborazanyl)amine‐borane (**19’**, Scheme 6), but based on the work of Baker, can now be attributed to B‐(cyclotriborazanyl)amine‐borane (**19**) with the other product formed being **1**. The mechanism for this reaction was proposed to be ‘off‐metal’, Manners observed the formation of R_2_N=BH_2_, with this being isolable for diisopropylamine‐borane (**16**, Scheme 5), during the reactions which could then go on to form cyclic/polymeric materials.

**Scheme 7:**
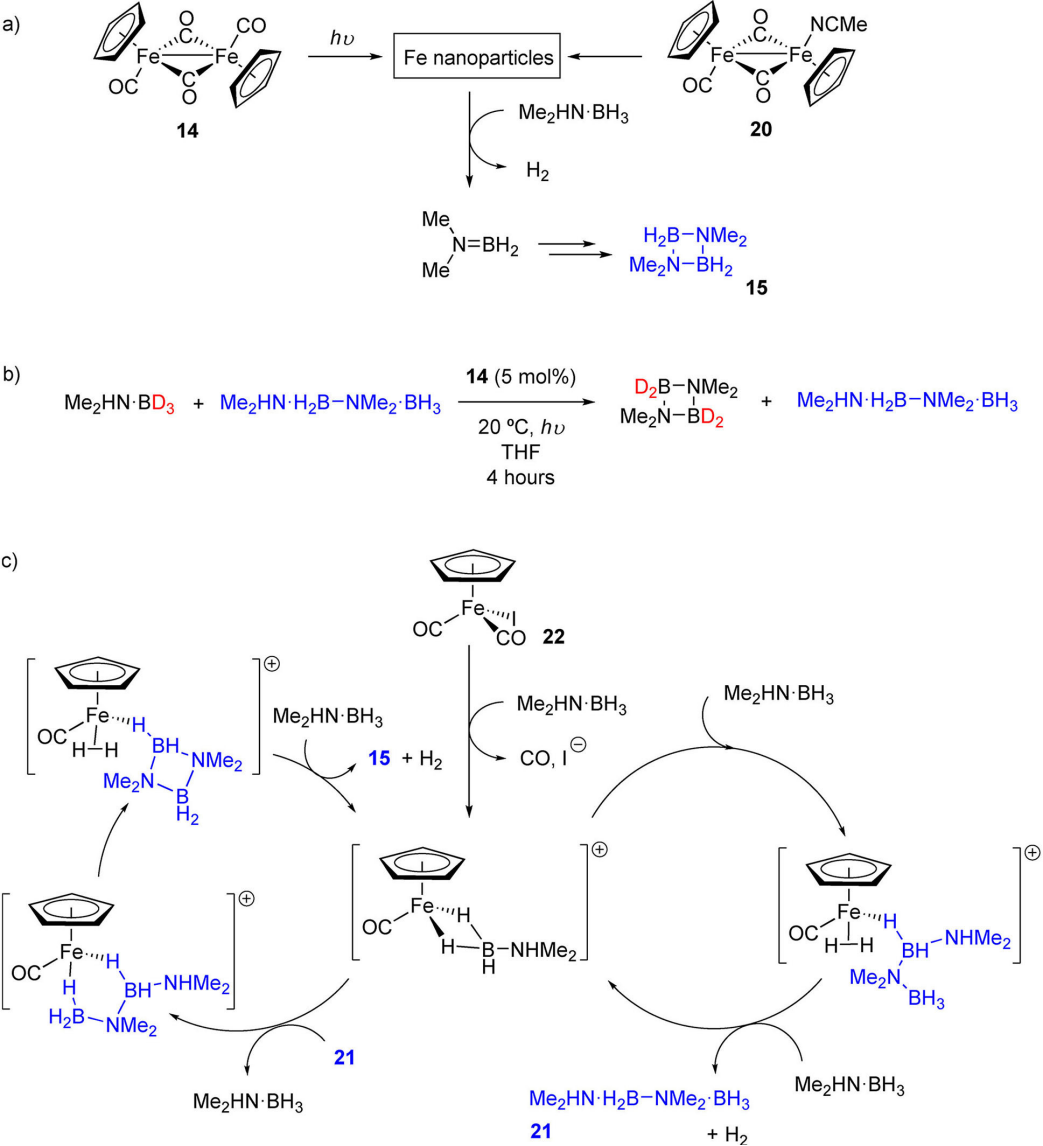
a) Iron carbonyl dimers form nanoparticles which undertake DHC, with the amino‐borane product (Me_2_N=BH_2_) being cyclized off‐metal. b) In contrast the mononuclear iron complex **22** appears to be homogeneous. c) Proposed reaction mechanism using **22**. In both reactions, photoactivation takes place using a medium pressure Hg lamp.

In a detailed follow‐up study, Manners further investigated the mechanism of cyclodimerization to form product **15**.^20^ Two different reaction mechanisms were found to be at play depending on the choice of pre‐catalyst. Catalyst **14** and catalyst **20**, a stable complex which is formed on photo‐irradiation of **14** in MeCN, were both shown to form nanoparticles, which facilitate the dehydrocoupling *via* Me_2_N=BH_2_, which forms in an iron catalyzed process but then cyclizes to form **15** off‐metal (Scheme 7a). Pre‐catalysts **14** and **20** did not lead to cyclization of telomer **21** and this dehydrocoupled intermediate was not formed in any great quantity during catalysis. As demonstrated by Manners,^19^ a cross‐over type experiment is the ideal method to check for the likelihood of Me_2_N=BH_2_ or **21** being an intermediate, even if it is not observed during catalysis (Scheme 7b). Here, deutero starting material and **21** were reacted under catalytic conditions with **14**. Deutero‐**15** was observed and unreacted **21**, with no cross‐over obtained. This confirms that the reaction catalyzed by **14** does not involve **21** as an intermediate.

**20** is highly active and did not need photoactivation in order for the reaction to proceed to completion in 20 minutes. The heterogeneous nature of the reaction mixtures was confirmed using a range of techniques including TEM, DLS and synthetic probes, such as reaction poisoning using PMe_3_. PMe_3_ is a useful test for heterogeneity since sub‐catalytic amounts of phosphine should coat all the active sites on a heterogeneous surface and thus prevent catalysis taking place.^21^ In contrast, pre‐catalyst **22**, which also needs photo‐irradiation, was believed to be homogeneous in nature, but the reaction was shown to proceed *via* linear telomer **21** in a two‐step reaction mechanism (Scheme 7c).

**Figure 3:**
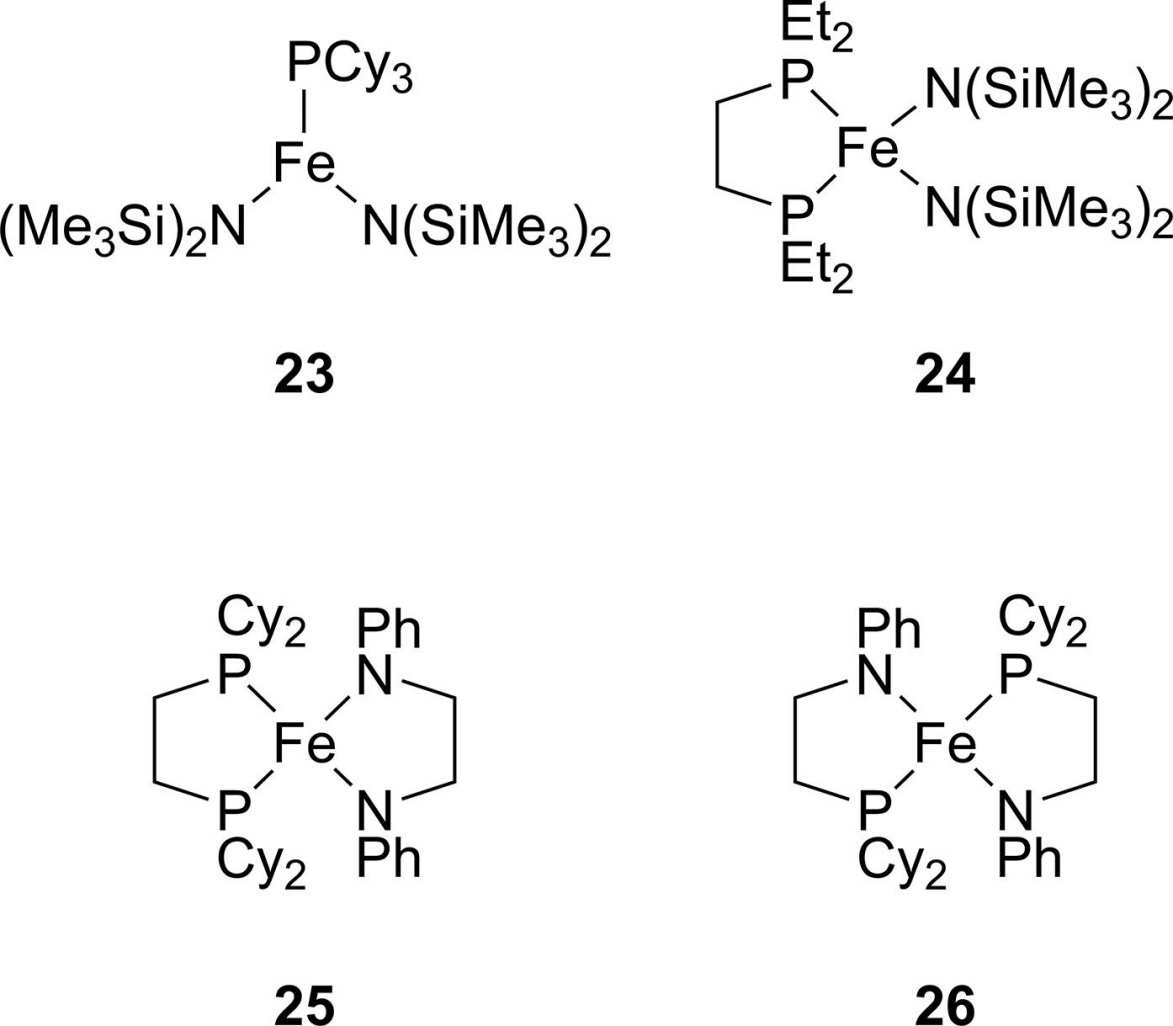
Complexes used by the Baker group in their publication in 2012.

Following on from their pioneering work on ammonia‐borane DHC using nickel, which as previously mentioned also included the earliest attempt at catalysis with iron,^9^ Baker and co‐workers developed a series of iron‐amido complexes (**23**–**26**, Figure 3) that showed activity for the DHC of ammonia‐borane.^22^ The authors synthesized the series of iron(II) complexes, starting with a mixed monodentate *P*‐ and *N*‐ ligand system (**23**) and progressing through to mixed amido‐phosphine bis‐chelating ligands (**25** and **26**). The pre‐catalysts operate at room temperature but for full conversion to occur the reactions were heated to 60 °C. THF or diglyme was also used as the reaction solvent, likely due to the low solubility of substrate in, for example, hydrocarbon solvents. Measurement of the H_2_ released showed that between 1 and 1.7 equivalents per ammonia‐borane could be formed (pre‐catalysts **25** and **23** respectively).

The reaction pathways for catalysis mediated by **23**, **24** and **26** were thought to be similar. The authors note that solutions darken and, in the case of **23** and **24**, protonated amido ligand is released, overall indicating that Fe(0) species have formed. The authors also note during more detailed studies with **23** that addition of two extra equivalents of PCy_3_ helps to stabilize the iron catalyst and generate turnover. However, the active iron complex could not simply be bulk metal because a commercial sample of Fe(0) with and without additional PCy_3_ did not lead to product formation. Interestingly, when a stoichiometric mixture of **23** and ammonia borane was allowed to crystallize over the course of several weeks an Fe_9_ cluster, which was supported by PCy_3_ ligands, was obtained.

**Figure 4:**
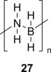
Structure of poly(aminoborane).

**25** is a highly active DHC catalyst, where a 5 mol % solution shows complete loss of starting material in 15 minutes and even when the loading was reduced to 1 mol % complete uptake of starting material took place in 2 hours. In this case the black precipitate formed during catalysis was completely inactive, with no further reaction observed when an extra 20 equivalents of ammonia‐borane were added. The product of reaction with **25** was poly(aminoborane) (**27**, Figure 4). The other complexes tested displayed little selectivity for the formation of the polymeric product, with a mixture of poly(aminoborane) (**27**) and poly(borazylene) (**9**) being present. Production of poly(borazylene) is, arguably, most desirable simply because the substrate has been dehydrocoupled multiple times, releasing multiple equivalents of H_2_ per equivalent of substrate used.

**Figure 5:**
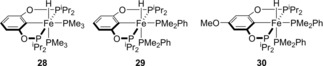
Structure of the complexes used by Guan.

**Scheme 8:**
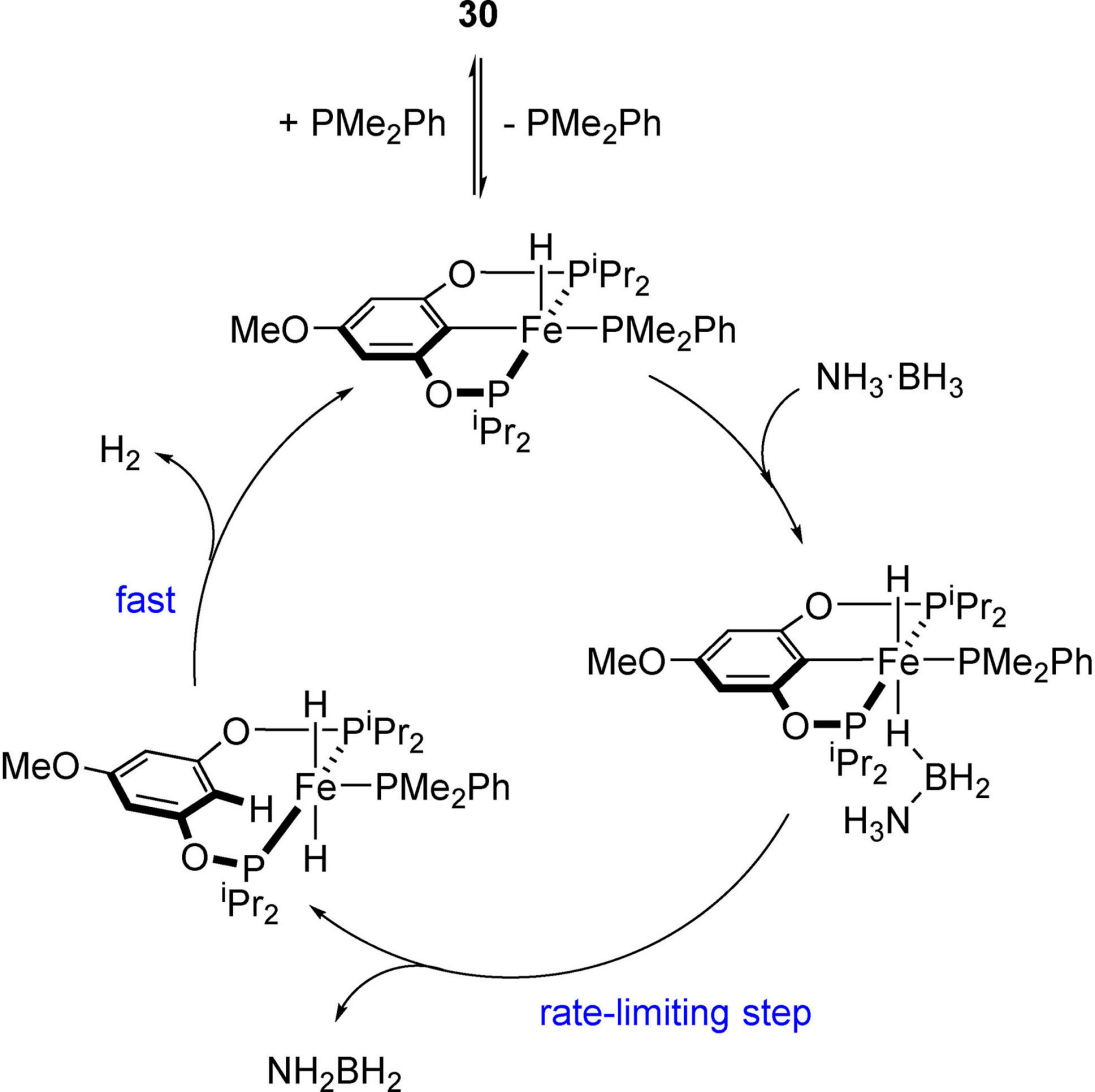
Proposed catalytic cycle for DHC using pre‐catalyst **30**.

Guan and co‐workers have also demonstrated that iron POCOP pincer complexes (Figure 5) show activity for ammonia‐borane DHC.^23^ This study demonstrated how changes in sterics of the ligands as well as the electronic properties can change the reactivity of a catalyst. The authors found that increasing the steric bulk from trimethylphosphine (**28**) to dimethylphenylphosphine co‐ligand (**29**) gave an increase in the rate. The POCOP pincer was then altered by adding a donating methoxy substituent to the *para* position (**30**) to increase the electron density at iron, not only facilitating the loss of PMe_2_Ph to form the active catalyst, but also helping to stabilize the coordinatively unsaturated metal center and thus helping to further increase the reactivity. Although ammonia‐borane DHC can take place in the absence of catalyst at 60 °C, the rate of reaction is much less than that obtained with the POCOP pre‐catalysts. Detailed mechanistic analysis using **30** showed a homogeneous reaction with first order dependence on pre‐catalyst and zero order dependence on substrate. KIE studies also indicated a rate‐limiting step which involved simultaneous cleavage of both N−H and B−H bonds, whilst the release of more than two equivalents of H_2_ per molecule of H_3_N⋅BH_3_ indicated that NH_2_BH_2_ was released from the metal center during catalysis. This latter point was backed up experimentally through the use of cyclohexene as a trapping reagent (H_2_N−B(C_6_H_12_)_2_ was observed at 47.8 ppm in the ^11^B NMR). Overall, a catalytic cycle could be proposed (Scheme 8).

**Scheme 9:**
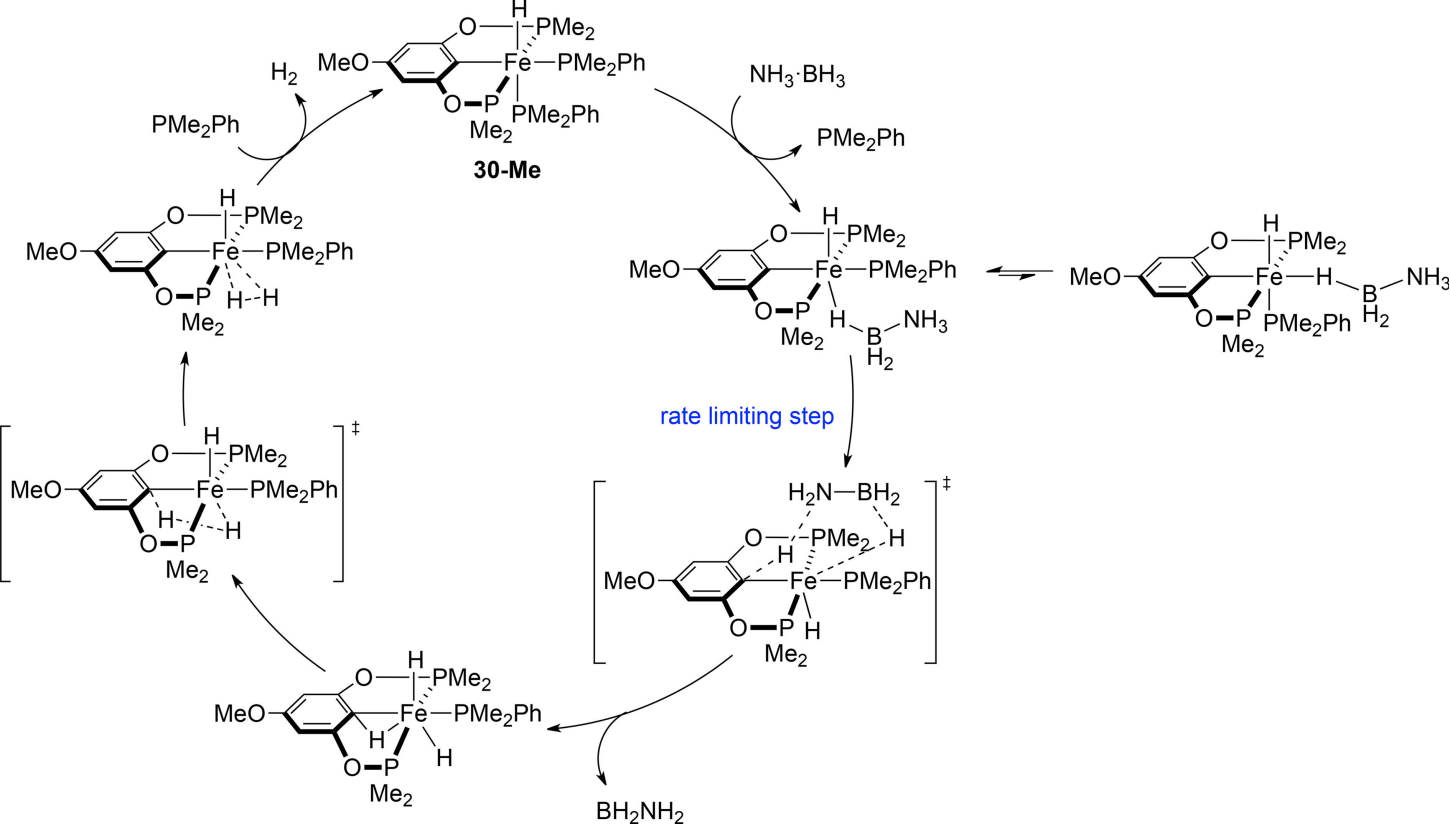
Liu and Wang's postulated catalytic cycle from DFT calculations.

Liu and Wang subsequently used DFT calculations to provide further weight to the aforementioned Fe‐POCOP catalyzed ammonia‐borane dehydrocoupling mechanism.^24^ Using a slightly simplified ligand set, where the ^i^Pr groups of **28**, **29** and **30** were replaced by Me (i. e. **28‐Me**, **29‐Me**, **30‐Me**), calculations were performed which considered the likelihood of the cooperativity of the ligand in the overall catalytic process. Comparing the three iron species, complex **30‐Me** was shown to be the most active for ammonia‐borane where the energy barrier for the rate‐determining step was calculated to be 17.6 kcal mol^−1^ (compared to 24.5 and 22.4 kcal mol^−1^ for complexes **28‐Me** and **29‐Me**), in‐line with the original experimental results. A catalytic cycle was proposed (Scheme 9) which, again, is in good agreement with that proposed by Guan.

**Figure 6:**
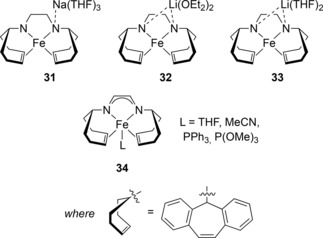
Structure of the Fe(I) amido complexes and Fe(0) complex (**34**) used by Grützmacher and co‐workers.

Grützmacher and co‐workers have used an Fe(I) amido olefin catalyst for DHC of dimethylamine‐borane.^25^ No reaction was observed when H_2_ was reacted with **31**, indicating the need for a substrate that contained protic and hydridic character, such as an amine‐borane. To compare counter ion effects both the Na (**31**, Figure 6) and Li (**32** and **33**) complexes were compared and tested in catalysis. **31** was the most active catalyst where, not only was it possible to obtain complete DHC of dimethylamine‐borane in 4 h at room temperature with 5 mol % pre‐catalyst, but three sequential additions of 20 equivalents of substrate over 12 h gave efficient DHC. Moreover, they also provided a rare example of intermolecular DHC of silanes with alcohols to make poly(alkyl silyl ethers) at RT. During the DHC of dimethylamine‐borane the authors observe the formation of the telomer intermediate (**21**), which is postulated by both Grützmacher and Manners to be indicative of a homogeneous mechanism. This was backed‐up by SEM analysis and addition of PPh_3_ or P(OMe)_3_ to the reaction mixture, where although rate of reaction decreased, full conversion was still obtained. Use of THF as the solvent or addition of reagents to sequester the cation (15‐crown‐5 or [(^n^Bu)_4_N]Br) led to a drop‐off in yield and/or slight slowing of the reaction.

Grützmacher then synthesized Fe(0) complexes using a similar ligand to their previous work (of the form **34**, where L=THF or MeCN, Figure 6).^26^ In this study they expanded the substrate scope, using methylamine‐borane and ammonia‐borane to demonstrate that the new catalyst was incredibly active, able to catalyze the formation of polymeric materials within minutes at room temperature. Even at 1 mol % loading, 95 % conversion of methylamine‐borane was observed in 20 minutes. Interestingly, **31** is not active in methylamine‐borane and ammonia‐borane DHC. Sub‐catalytic loading of P(OMe)_3_ to the methylamine‐borane DHC reaction catalyzed by **34**⋅THF reduces the rate but complete conversion is observed after 11 hours. Addition of 2 equivalents of phosphite per Fe‐center leads to only 63 % product after 15 hours (whereas an additive‐free reaction is complete in around 8.5 minutes). These studies suggest that the reaction is homogeneous in nature, but that the phosphite adduct **34**⋅P(OMe)_3_ is not an active catalyst.

In contrast, ammonia‐borane DHC necessitates catalysis at 5 mol % loading and a longer reaction time, although this is still highly competitive with complete conversion observed in 5 hours at RT using **34**⋅THF. Poisoning experiments suggest that a heterogeneous reaction is at play, although detailed mechanistic insight is needed for both homo‐ and heterogeneous reactions in order to unravel the modes of reactivity possible with these highly active iron complexes.

**Scheme 10:**
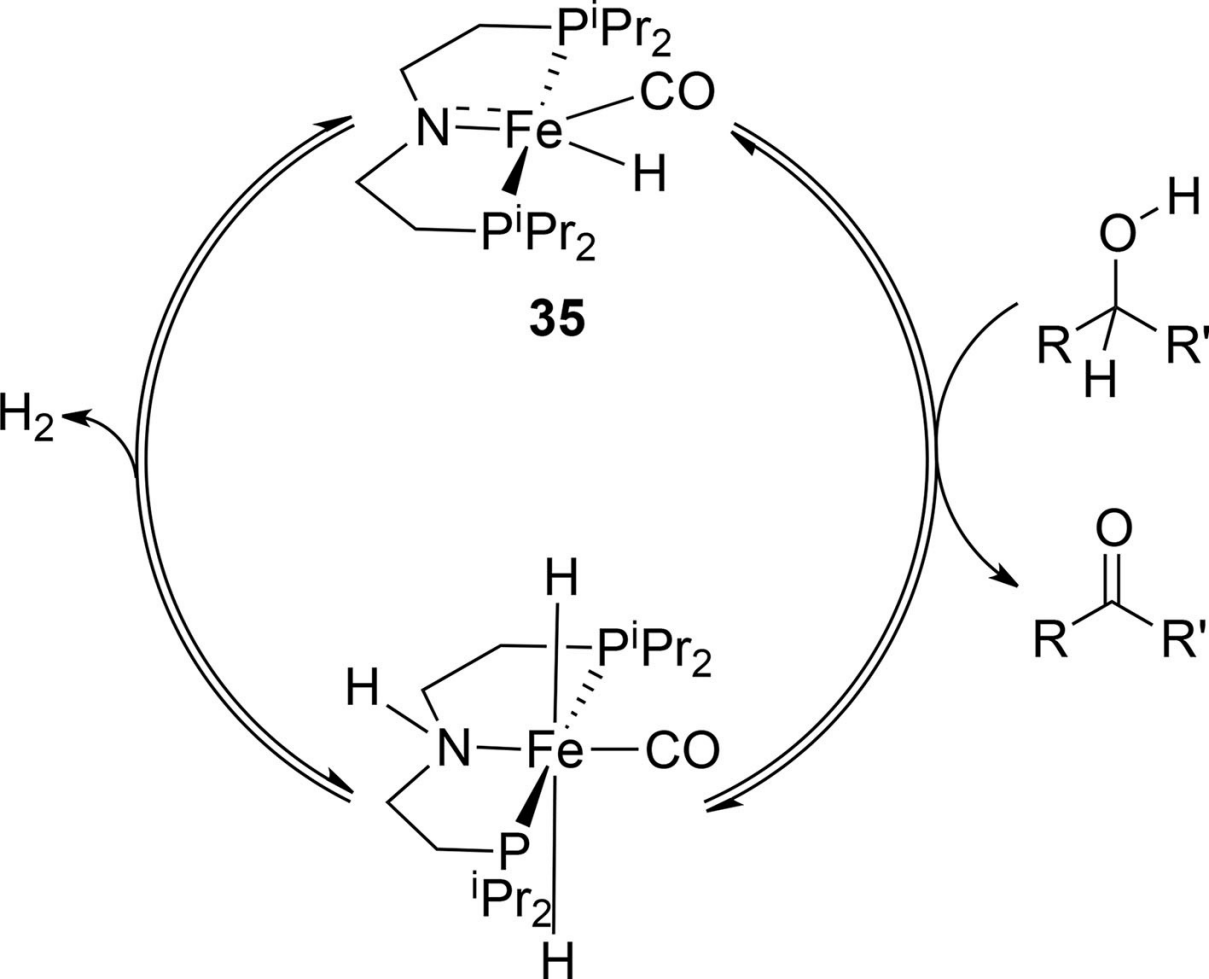
Catalytic cycle for the dehydrogenation of alcohols with catalyst **35** proposed by Schneider.

Schneider and co‐workers have shown very effective DHC activity with a pre‐catalyst containing a PNP ligand (**35**, Scheme 10).^27^ Iron PNP catalysts have previously been used in the dehydrogenation of methanol and water to produce CO_2_ and H_2_ by Beller in 2013 and the catalyst used in this DHC study was previously used for acceptorless dehydrogenation/hydrogenation of alcohols and ketones and dehydrogenation of formic acid (Scheme 10).^28^

**Scheme 11:**
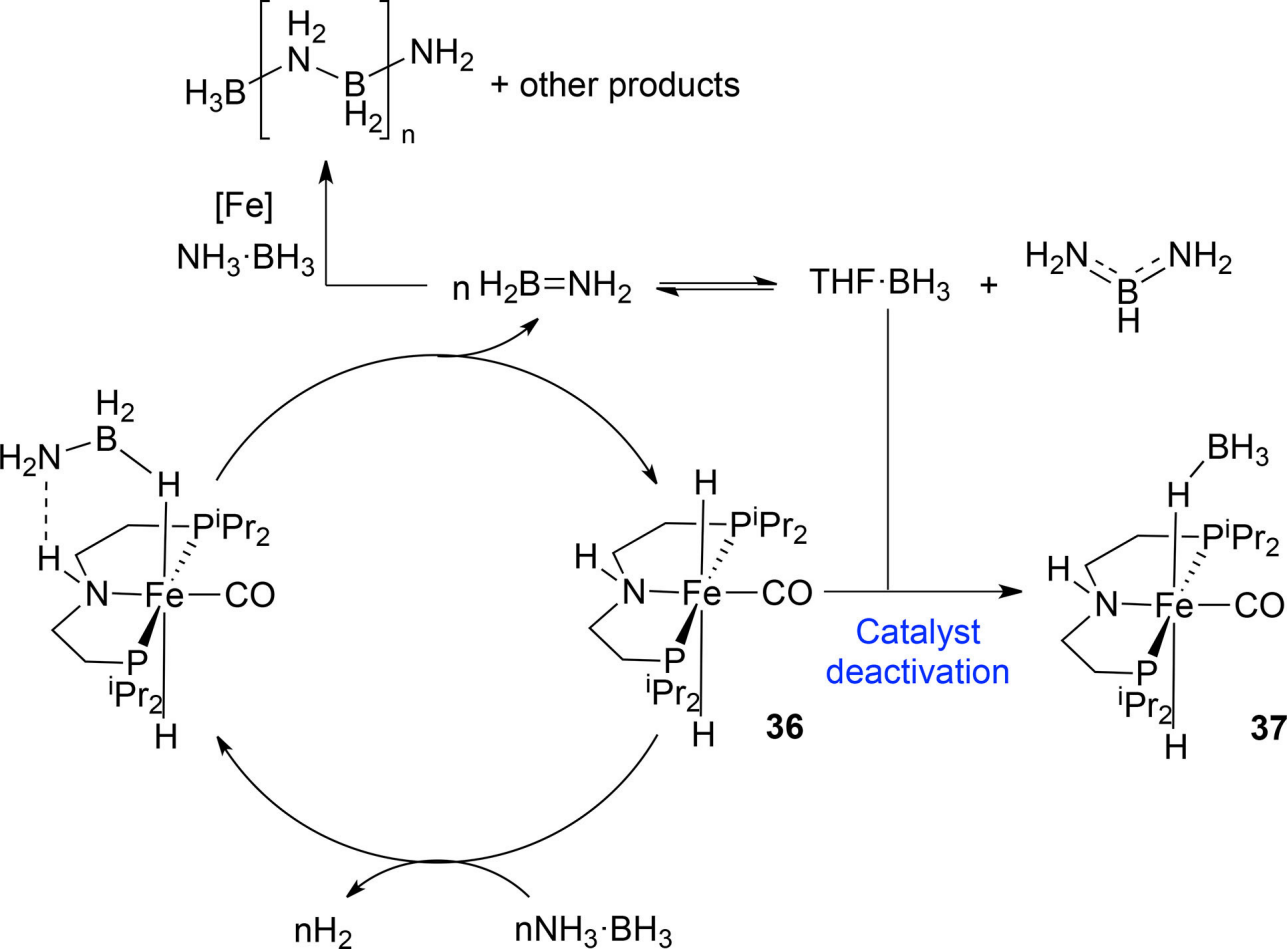
Schneider's ammonia‐borane DHC catalytic cycle including catalyst deactivation product, **37**.

Schneider and co‐workers obtained complete conversion of NH_3_⋅BH_3_ with catalyst loadings as low as 0.5 mol % at room temperature, which is the lowest loading to date for an iron catalyst. Even at 0.1 mol % a turnover number (TON) of 95 was achieved. Detailed mechanistic understanding showed that catalyst deactivation was likely to be occurring *via* BH_3_ coordination to the *trans*‐dihydride resting state **36**. Addition of a catalytic amount of NMe_2_Et limited formation of the inactive complex, **37**, by trapping BH_3_ and thus, at a catalyst loading of 0.2 mol % with 0.8 mol % NMe_2_Et, a TON of 330 was achieved. With knowledge of the catalyst deactivation route, addition of NMe_2_Et to the analogous ruthenium catalyzed reaction led to the TON increasing by a factor of three compared to the original reported TON.^29^ The reaction is not selective, resulting in a large number of products, including **1**, **5**, **9**, **19** and **27**. The mechanism proposed involves the ligand exhibiting somewhat non‐innocent behaviour, assisting with N‐ and B‐atom coordination (Scheme 11).

**Figure 7:**
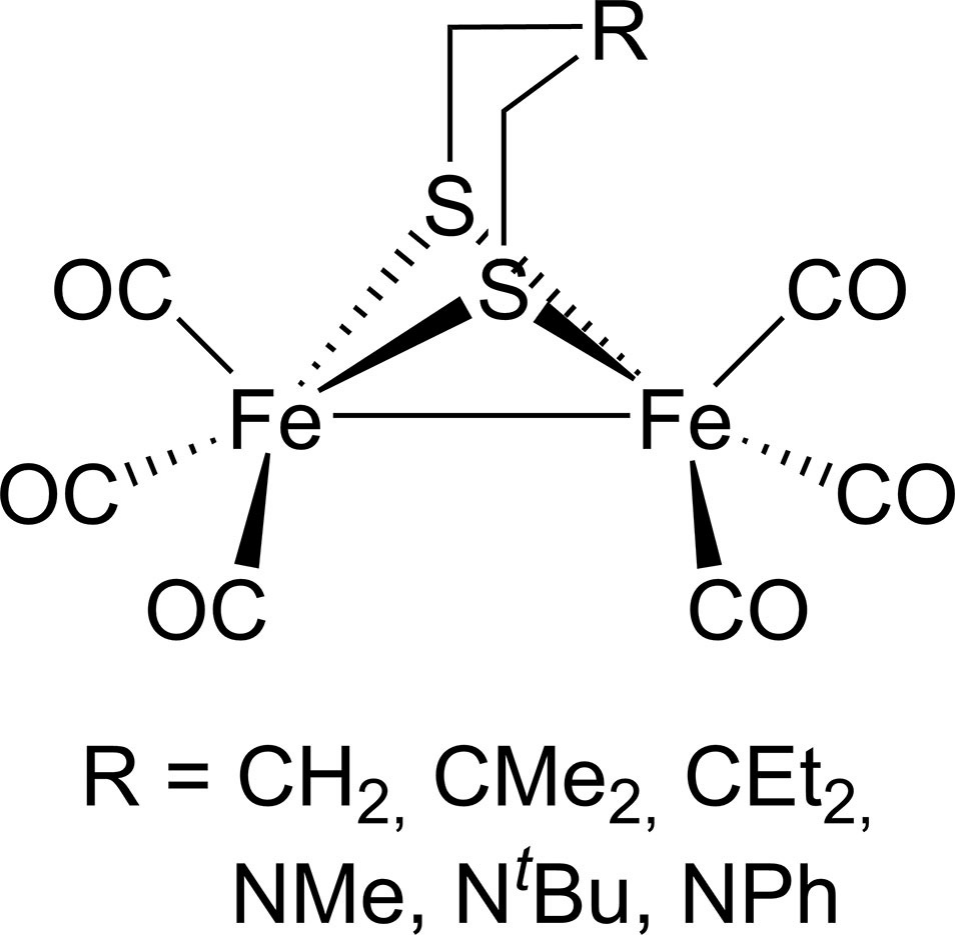
The range of diiron complexes used in the study by Darensbourg and Bengali.

One of the most recent examples of iron–catalyzed DHC was reported by Darensbourg, Bengali and co‐workers.^30^ The authors synthesized a number of diiron pre‐catalysts with varying bridgehead substituents (Figure 7) and used them as photocatalysts to dehydrocouple Me_2_HN⋅BH_3_ with the greatest rate of reaction obtained when R=CH_2_ (complete H_2_ evolution was observed with 10 mol % catalyst loading after only 85 minutes of photolysis at RT). The product of this DHC reaction is the four membered cycle **15**. Extensive mechanistic studies, including DFT calculations and kinetic investigations, were also undertaken using Et_3_N⋅BH_3_ as a model system. Firstly, photolysis of the complexes with UV light led to loss of CO and the formation of a vacant site at one of the Fe‐centers, therefore the complex is primed to coordinate reagent and undertake catalysis. DFT studies showed that agostic interactions are likely to play a role in stabilizing this vacant site and that this ‘diminishes the residence time of the substrate’ in that it leads to rapid release of substrate from the metal center when using P(OEt)_3_ as a ligand which competitively coordinates to the iron center, displacing Et_3_N⋅BH_3_. However, the rate of H_2_ release from Me_2_HN⋅BH_3_ is inversely related to the ability to form agostic interactions, hence the fastest dimethylamine‐borane DHC reaction being observed for R=CH_2_. It should also be noted that the ultimate combination of residence time (ability to coordinate substrate and thus undertake reaction) and removal of proton and hydride from the Me_2_HN⋅BH_3_ substrate in a heterolytic process is likely to be when R=NMe_2_. The biomimetic nature of this particular complex is highlighted by the authors through the similarities to the iron hydrogenase enzyme. The reaction is likely to be homogeneous in nature, but the authors state that ‘a dark brown/black decomposition solid was also deposited on the sides of the flask’; there is a possibility that this reaction is actually heterogeneous since Manners had previously shown nanoparticles can be generated *via* irradiation of iron carbonyl pre‐catalysts. No tests on catalytic competency were carried out on this precipitate, but presumably this is a minor component and simply the end‐point for some of the complex after catalysis has taken place, especially since irradiation throughout the dimethylamine‐borane DHC reaction was needed in order to afford the product.

**Scheme 12:**
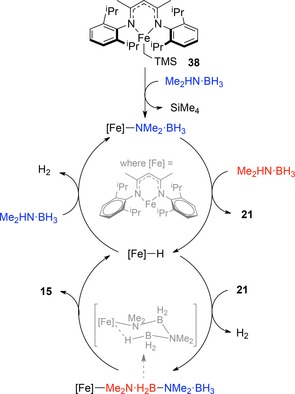
Postulated catalytic cycle for dimethylamine‐borane DHC using **38**.

Our own research has focused on the use of Fe(II) which can undergo σ‐bond metathesis type chemistry to furnish a completely redox‐neutral catalytic cycle. Relying on the proton‐donor/hydride‐donor nature of amine‐boranes, where the amine delivers protons and the borane component delivers hydrides, we should be able to access a catalytic cycle which involves sequential iron‐amido/iron‐hydrido intermediates. Pleasingly, in terms of substrate scope, amine‐boranes react under mild conditions, with Me_2_HN⋅BH_3_, ^i^Pr_2_HN⋅BH_3_ and BnMeHN⋅BH_3_ all undergoing complete conversion to dehydrocoupled product in 3 to 12 hours at room temperature with 1 mol % **38**, and are thus suitable for mechanistic investigation.^31^ Unfortunately ammonia‐borane was not a suitable substrate for DHC simply due to lack of solubility in the reaction solvent (C_6_D_6_) and the poor long term stability of the pre‐catalyst in other standard solvents for ammonia‐borane DHC e. g. THF, diglyme, meaning our scope for DHC is limited. Our mechanistic studies showed that the reaction appears to be first order in both substrate and pre‐catalyst, but with saturation‐type kinetics taking place at higher substrate concentrations. Isolation of a chair‐like complex, believed to be the catalyst resting state, along with an iron hydride dimer led us to postulate a catalytic cycle (Scheme 12) which involves the release of **21**, synonymous with a homogeneous reaction which was backed‐up by poisoning studies.

## 3 Phosphine‐Borane Dehydrocoupling

**Scheme 13:**
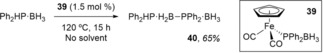
The complex used by Manners and co‐workers with optimized conditions for diphenylphosphine‐borane DHC.

In 2008 the Manners group published work on DHC of diphenylphosphine‐borane using a piano‐stool complex (**39**, Scheme 13).18a The group showed that the CO ligands could be displaced by PMe_3_, an indication that activation by phosphine‐borane reagent was possible. The DHC of Ph_2_HP⋅BH_3_ required relatively harsh conditions (120 °C in the absence of solvent) and the product obtained was a telomer (**40**) rather than a cyclic trimer, which would be obtained when the compound is fully dehydrocoupled. However, the reaction had reached 65 % conversion within 15 hours using 1.5 mol % **39**. The reaction was poor when undertaken in solvent and use of UV irradiation did not facilitate catalysis.

Use of the iron dimer Fe_2_(CO)_9_ at 1.5 mol % loading, neat at 120 °C also gave **40**, but with slightly increased conversion of 80 %. However, use of lower reaction temperature (60 °C) or in toluene at 60 or 110 °C did not give appreciable amounts of product.

**Figure 8:**
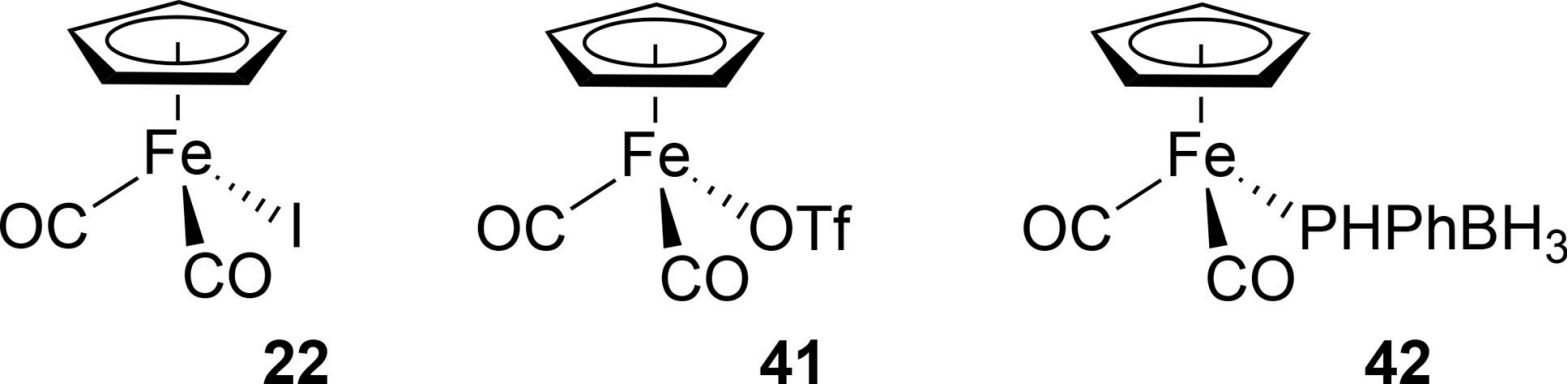
Structure of the pre‐catalysts used for the polymerization of phosphine‐boranes.

**Scheme 14:**
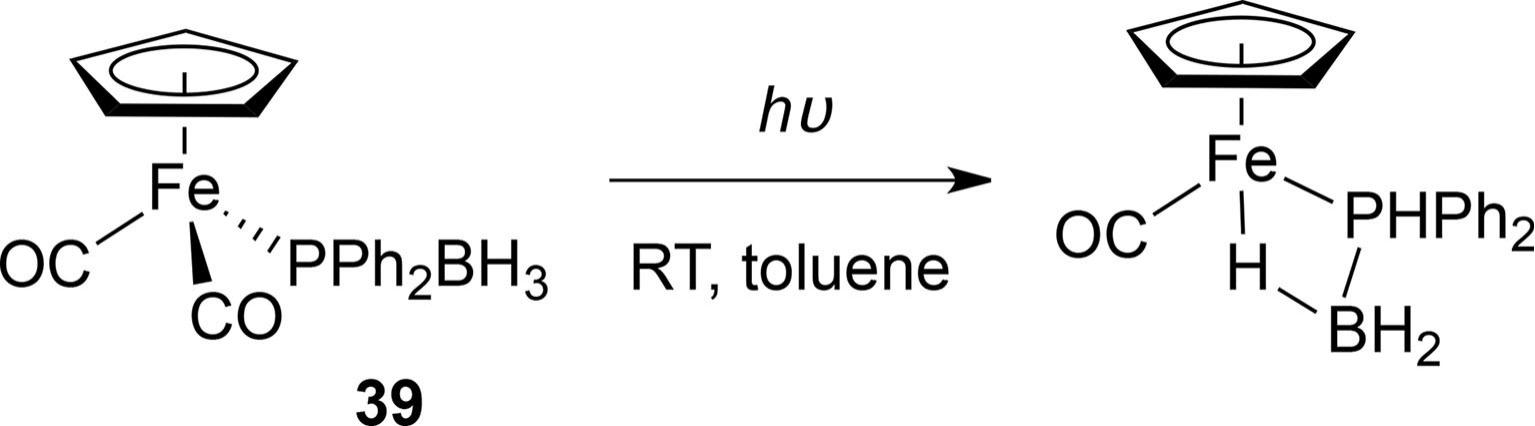
An intramolecular iron‐hydride interaction was observed when **39** was irradiated.

The FeCp system was subsequently adapted for the synthesis of high molecular weight poly(phosphine‐boranes).18b Initially it was shown that the iodide adduct **22** (Figure 8) gave complete conversion of phenylphosphine‐borane at 10 mol % loading after 24 h at 100 °C. The molecular weight for the polymer product was modest (*M*_n_=18,000 g mol^−1^, PDI=2.0), whereas when the iodide was changed for a less coordinating triflate (**41**) high molecular weight polymer (*M*_n_=59,000 g mol^−1^, PDI=1.6) was obtained under identical reactions conditions, but with a lower loading of pre‐catalyst (1 mol %). Reaction of **22** or **41** with one equivalent of phenylphosphine‐borane led to the phosphido‐borane adduct **42**, which could also be employed in dehydropolymerization (*M*_n_=80,000 g mol^−1^, PDI=1.6 at 1 mol % **42**, toluene, 100 °C, 24 h). Control of the molecular‐weight using **41** was also demonstrated by varying the catalyst loading, with the results being consistent with a chain‐growth mechanism: at higher catalyst loading lower *M*_n_ and *M*_w_ were observed due to more propagating chains being formed. Similar to the previous study, it was postulated that loss of CO occurred, allowing coordination of a molecule of phenylphosphine‐borane, DFT studies backed this up and isolation and crystallization of a mono‐carbonyl species provided some evidence for this (Scheme 14).

**Scheme 15:**
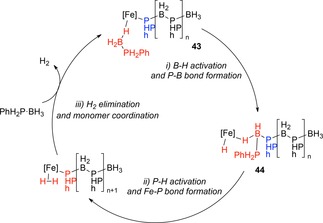
Manners’ catalytic cycle for phosphine‐borane DHC.

Overall, a reaction mechanism was proposed which involved coordination of the hydride of the phosphine‐borane (**43**) to the iron center of the phosphido‐borane intermediate. Insertion of the borane to form a new P−B bond (**44**) followed by P−H activation to release H_2_ and generate an Fe−P bond completes the catalytic cycle (Scheme 15).

**Scheme 16:**
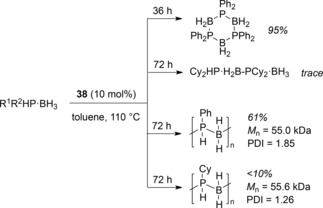
Phosphine‐borane DHC using Fe(II) β‐diketiminate complex **38**.

The activity of **38** in amine‐borane DHC also encouraged us to explore phosphine‐borane DHC.^31^ Unfortunately the reaction conditions necessary for phosphine‐borane DHC are such that we have only been able to explore substrate scope (Scheme 16) and undertake preliminary mechanistic investigations in the form of radical trap and poisoning studies. These latter investigations suggest that the reaction is homogeneous in nature and that radicals are not involved in catalysis. Poly(phosphine‐boranes) can be formed, but note for cyclohexylphosphine‐borane, although all of the starting material was consumed in the reaction and high molecular weight polymer can be formed, this is very much the minor component of the reaction, with short chain oligomers (*M*_n_<2000 g mol^−1^) being the major products.

## 4 Phosphine Dehydrocoupling

**Scheme 17:**
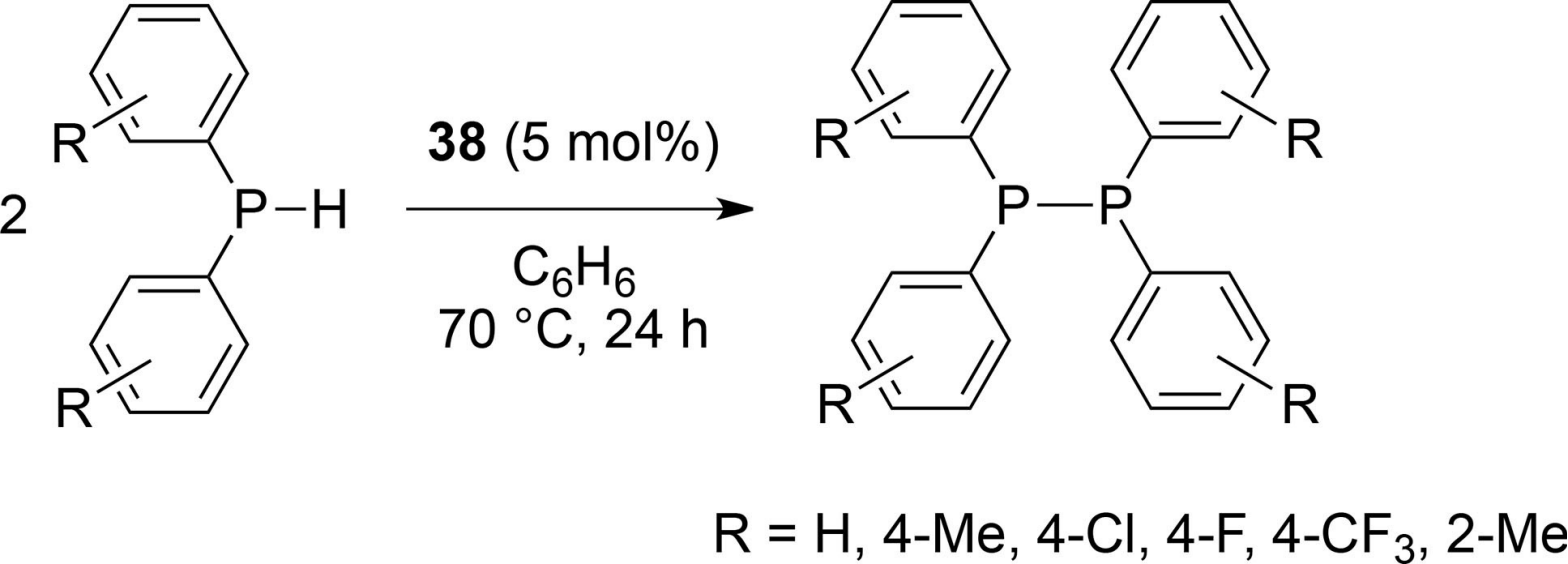
Dehydrocoupling of secondary phosphines using pre‐catalyst **38**.

Homodehydrocoupling of group 15 compounds catalyzed by iron has not been as well explored as heterodehydrocoupling. However, research from our own group has shown that iron(II) β‐diketiminate complex **38** can be used to dehydrocouple secondary aryl phosphines with relative ease (Scheme 17).^32^ Spectroscopic yields range from 100 % (85 % isolated yield) when R=H, through to 36 % (no product isolated) when R=2‐Me; the lack of reactivity with the latter substrate is presumably limited by steric hindrance. Likewise, a mixture of sterics and electronics limit the reactivity of 4‐OMe, 4‐NMe_2_ and 2‐OMe substrates with a reaction temperature of 100 °C being necessary and spectroscopic yields varying between 33 %, 100 % and 10 % respectively. Similarly, Cy_2_PH, CyPH_2_ and PhPH_2_ are very challenging substrates, requiring more forcing conditions (e. g. 10 mol % **38**, 120 °C, 72 h) which leads to mixtures when the primary phosphines are employed. Unfortunately, stoichiometric reactions between phosphine and pre‐catalyst failed to yield any potential reactive intermediates, simply because dehydrocoupling is so facile, even at room temerature. However, radical trap and DFT studies suggested that the reaction was radical mediated: an interesting contrast to our amine‐borane and phosphine‐borane chemistry which merits further study.

## 5 Summary and Outlook

It is clear that a range of iron pre‐catalysts can be used to dehydrocouple both amine‐ and phosphine‐boranes. There are several mechanistic pathways observed for these catalysts with many bearing similarities to pathways proposed for other transition metal catalysts. Divergent homo‐ and heterogeneous reaction pathways are known within similar classes of iron pre‐catalyst (most notably in the work of Manners and Baker). With iron readily forming nanoparticles, it is also important that when determining the mechanism both homogeneous and heterogeneous catalysis should be taken into account and investigated. The fact that divergent reaction mechanisms appear to be at play when comparing homo‐ to heterogeneous catalyzed DHC is intriguing and is an area that could be exploited. Overall with detailed mechanistic understanding catalysis can be used to its full potential: a good example being Schneider's observation of catalyst deactivation, where a simple amine additive switches on catalysis and vastly increases the TON. With mechanistic understanding and catalyst development, iron clearly has the ability to compete with leading transition metal catalyzed amine‐borane DHC reactions. Beyond ammonia‐borane DHC, elegant examples of iron salts undertaking DHC of novel liquid hydrogen storage materials have been put forward in the literature, for example the investigations of Liu and co‐workers. The tolerance of such substrates, beyond the simplest amine‐boranes, hints that iron catalysis could be employed in more diverse transformations, with potential applications in materials science. However, more development is needed in this regard; phosphine‐boranes, which have applications in ceramics for example, are vastly underexplored but the potential of iron catalysis in this area, and in the broader field of dehydrocoupling, is clear and shows exciting potential.

## Biographical Information

*Nathan T. Coles studied Chemistry with Medicinal Chemistry at the University of Warwick, England. He completed his MChem in 2015 where he worked under the supervision of Dr Adrian Chaplin for his final year project, focusing on synthesizing macrocyclic rhodium complexes. During his time at Warwick, Nathan also took part in two summer projects investigating the catalytic cleavage of N‐O bonds with Dr David Fox. He has since moved to the University of Bath to conduct a PhD under the supervision of Dr Ruth Webster, where he is looking into the mechanism and intermediates of iron catalyzed transformations involving main group elements, he is currently in his second year of study*.

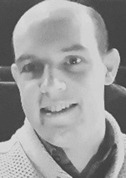

## Biographical Information

*Ruth L. Webster was born and raised in central Scotland and received her MSci degree from the University of Strathclyde, Glasgow, in 2007. She then moved to the University of Bristol to carry out her PhD under the supervision of Professor Robin Bedford. This research focused on Pd‐catalyzed C−H functionalization. In September 2011 Ruth was awarded a Government of Canada Commonwealth Research Fellowship with Professor Laurel Schafer at the University of British Columbia investigating group 4 initiators for ring‐opening polymerization. In October 2012 Ruth returned to the UK to take up a University of Bath Prize Fellowship in Catalysis, transitioning to Lecturer in 2014. The focus of her research is on the development of iron catalysts for the synthesis/manipulation of main group compounds*.